# Thermodynamically Consistent Linear Electroelastic Formulation and FEM Study of Patch-Actuated Smart Structures: Validation and Interface Stress Evaluation

**DOI:** 10.3390/ma19091864

**Published:** 2026-05-01

**Authors:** Mehmet Metin Ali Usal, Halil Özer

**Affiliations:** 1 Department of Mechanical Engineering, Kırklareli University, Kırklareli 39000, Türkiye; 2 Department of Mechanical Engineering, Yıldız Technical University, Istanbul 34349, Türkiye; hozer@yildiz.edu.tr

**Keywords:** constitutive equations, continuum electrostatics, field equations, finite element solution, piezoelectric, smart structure, 2D and 3D static analyses

## Abstract

In this study the electromechanical response of a cantilever composite beam with surface-bonded piezoelectric patches is examined, focusing on interface stresses that may initiate delamination. A thermodynamically consistent electroelastic framework was specialized to the linear piezoelectric law used in finite element software, and a two-dimensional (2D) finite element model was developed and validated under static actuation. The predicted tip displacement was compared against the analytical Euler–Bernoulli solution across all seven mesh levels of the convergence study; findings indicated that the converged ANSYS 17.1 result (h = 5 × 10^−5^ m) differed from the analytical value by 5.8%, a discrepancy attributed to the plane-strain assumption and the neglect of shear deformation in the Euler–Bernoulli formulation. To resolve the delamination-critical behavior, three-dimensional (3D) models were built using SOLID185/SOLID5 and SOLID186/SOLID226 elements. Interfacial peel σ_y_ and shear τ_xy_ stresses were evaluated along lengthwise (PATH1) and transverse (PATH2) paths at the patch–core interface, with maximum interface stresses occurring along the transverse PATH2 near the free end, where strong three-dimensional edge effects developed. Both element sets predicted a similar tip displacement, but the SOLID186/SOLID226 elements yielded peak interface stresses approximately 19% higher in peel and 87% higher in shear along the critical transverse PATH2. These findings demonstrate that element choice minimally affects global stiffness but significantly influences local interface stress prediction, providing practical guidance for the selection of appropriate models when assessing the delamination risk in piezoelectric-actuated composite beams.

## 1. Introduction

Surface-bonded piezoelectric patches are among the most practical tools available for the active control of composite beam structures. By exploiting the converse piezoelectric effect—whereby an applied electric field produces mechanical strain—these patches can generate bending moments, suppress vibrations, and control shape without adding significant mass to the host structure [1,2,3,4,5,6]. Their widespread use in aerospace, civil, and biomedical engineering has led to a growing body of work centered on modeling their electromechanical behavior [7,8,9,10,11].

When a piezoceramic patch is bonded to the surface of an elastic beam and subjected to electric potential, the mismatch in mechanical properties between the patch and the substrate generates localized interfacial stresses—peel stress, $\sigma_{y}$, acting normally on the interface, and shear stress, $\tau_{xy}$, acting tangentially. These stresses are largely invisible to global performance metrics such as tip displacement, yet they are the primary drivers of delamination failure, which is one of the most common and damaging degradation mechanisms in piezoelectric composite structures [12,13,14]. Despite this significance, the sensitivity of these interface stresses to finite element modeling choices—in particular, to the choice of element type and the completeness of the underlying theoretical framework—has received comparatively little systematic attention in the literature.

Lead–zirconate–titanate (PZT) piezoceramics, and, especially, PZT-5H, are the most widely used active materials in such configurations due to their high piezoelectric coupling coefficients and well-characterized electromechanical properties [15,16,17,18,19]. Beyond piezoelectric transduction, thermoelectric materials—which directly convert thermal gradients into electrical energy—represent a complementary and rapidly developing class of energy-harvesting smart materials [20], further broadening the context of multi-physics energy conversion in structural applications. In the present work, however, the focus is on static piezoelectric actuation, where the choice of finite element formulation has a direct and quantifiable effect on the predicted interface stress state.

From a theoretical standpoint, the standard linear piezoelectric constitutive law implemented in commercial finite element codes is almost universally used in practice; yet, its thermodynamic foundations are rarely made explicit. Several general electroelastic frameworks have been proposed in the literature [21,22,23], but the chain of assumptions that connects a full thermomechanical continuum formulation—one that admits body couples, asymmetric stresses, and finite deformation—to the symmetric-stress, small-strain model actually implemented in codes such as ANSYS is typically left implicit. This lack of transparency makes it difficult to assess which physical effects are being neglected and whether the simplified model is appropriate for a given problem.

From a numerical standpoint, the effect of element type selection on global response quantities such as tip displacement is well-understood to be modest once mesh convergence is achieved. What is far less well-documented is the sensitivity of local interface stress predictions—particularly in the three-dimensional edge region at the lateral boundary of a patch—to the order of the element formulation. First-order elements capture bending and through-thickness gradients more coarsely than second-order elements, and this difference becomes consequential precisely in the regions where delamination is most likely to initiate.

The present study addresses both of these gaps and makes two distinct contributions. First, a thermodynamically consistent electroelastic framework is derived from first principles, beginning with electrostatic balance, thermomechanical conservation laws, and the second law of thermodynamics, and it is then rigorously specialized—through explicit simplifying assumptions—to the symmetric-stress linear piezoelectric constitutive law used in ANSYS. This derivation makes transparent exactly which physical effects (body couples, asymmetric stresses, and finite deformation) are neglected in the standard implementation, providing a thermodynamically sound basis for the numerical models that follow. Second, a systematic two-dimensional and three-dimensional finite element study is carried out for a cantilever aluminum beam with surface-bonded PZT-5H patches under a 10 V static actuation. The 2D model is validated against a closed-form Euler–Bernoulli analytical solution and is shown to capture the global electromechanical response accurately. The 3D models, built with two different element sets—first-order SOLID185/SOLID5 and second-order SOLID186/SOLID226—are then used to evaluate the interfacial peel and shear stresses along a lengthwise path (PATH1) and a transverse path (PATH2) at the patch–core interface. The results demonstrate that, while the element type has a negligible effect on tip displacement (<0.3%), it has a decisive effect on the predicted interface stress peaks, with the higher-order formulation yielding values up to 87% larger along the critical transverse path.

The paper is organized as follows: Section 2 presents electrostatic and thermomechanical balance equations. In Section 3, entropy production inequality is derived, and a constitutive framework is established via a Legendre transformation. Section 4 contains the constitutive and field equations and specializes them to a static linear piezoelectric case. Section 5 presents the 2D and 3D finite element models, the convergence study, analytical validation, and the results of interface stress. Section 6 provides a summary of the conclusions and offers practical recommendations for model selection in the delamination assessment.

## 2. Electroelastic Formulation

In this study, the statements for the electrostatic forces and moments affecting the polarized continuum are derived from the fundamentals of electrostatics in a dielectric medium. These electrostatic interactions are incorporated into the thermomechanical formulation, and the governing equations are derived from electrostatic and thermomechanical balance equations, along with the second principle of thermodynamics. In this work, the fundamentals of electrostatics in a dielectric medium are utilized to derive statements for the electrostatic forces and moments acting on the polarized continuum. These electrostatic forces and moments are incorporated into the thermomechanical formulation, and the governing equations are derived via the conservation principles of charge, mass, linear momentum, angular momentum, and energy, along with the second law of thermodynamics.

The entire electroelastic problem contains 22 unknowns. The unknown variables of the problem are density (one variable), velocity (three constituents), symmetric stress (six constituents), the electric field (three constituents), the polarization field (three constituents), heat flux (three constituents), temperature (one variable), entropy (one variable), and inner energy (one variable). The relationships for these unknowns are as follows: conservation of mass (one equation), —balance equations (a linear momentum equation and angular momentum equation—three force equations and three moment equations), conservation of energy (one equation), Gauss’ law for dielectric media (one equation), one constitutive relation, and Faraday’s law for dielectric media (three equations). In this study, a total of 13 equations are written. Because the number of unknowns exceeds the number of equations, additional equations are needed. These required equations are subsequently obtained as constitutive equations. Of the nine constitutive equations that bridge the gap, six are scalar components belonging to the symmetric stress tensor, and three are the scalar components of the polarization vector.

### 2.1. Motion and Deformation

**Figure 1:**
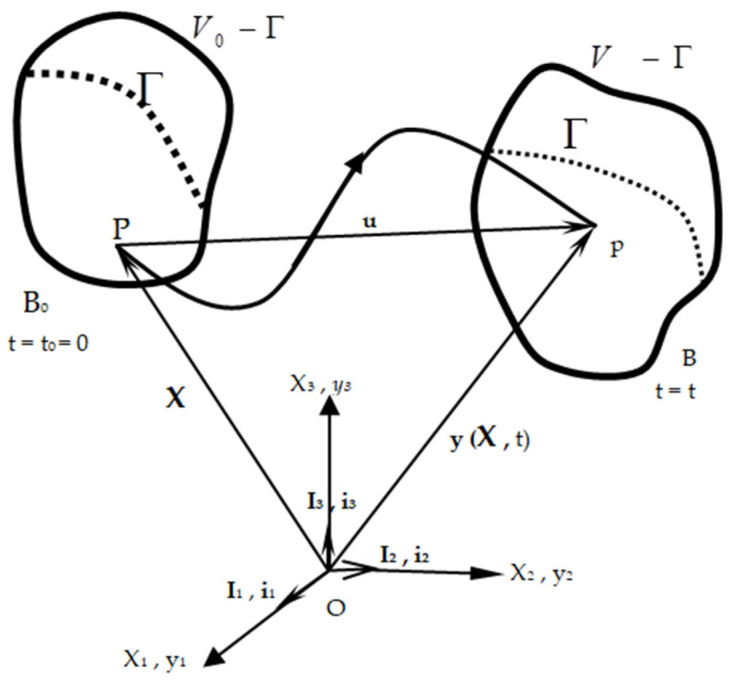
Movement and deformation in a continuous medium, and Γ discontinuity surface.

Here, the Cartesian coordinate set is selected to designate the position of the continuum in space. It is assumed that there is a discontinuous surface Γ(*t*) moving with speed u, in a finite continuum with volume V(t) and with surface S(t), (Figure 1). The y=y(X,t) function is used to indicate the motion of the medium. The deformation of the medium becomes significant as X is drawn near d X, and as y is drawn near d y, and as the connection between two neighbors occurs. This situation can be expressed with a transformation in the form of d y = F ⋅ d X, $\;y_{m}\;=\;y_{m}\;(X_{M}\;,\;t)$ and (M,m= 1, 2, 3). Here, the size of the $F=\;y_{m,M}i_{m}\;I_{M}$ is determined as long as the movement is given, and is called the deformation gradient [24,25].

Large indices (I, J, K, …) consistently represent the material (Lagrangian) coordinates $X_{I}$ throughout the text. Small indices (i, j, k, …) consistently represent the spatial (Eulerian) coordinates $y_{i}$.

### 2.2. Electrostatic Balance Equations

To ensure integrity in the study, local balance equations will be given as a summary. In a continuum with volume V(t) bounded by the surface S(t), a discontinuity surface Γ(*t*) moving with velocity u is considered [23,24]. The medium under investigation does not contain free volumetric charge distribution.

The body force and body couple distributed as a result of the interaction between the polarization and the electric field are expressed as follows:

(1) $$F_{P}=(P\cdot \nabla )\;E$$

(2) $$m_{P}=P\times E$$

In the interaction of the medium in question with its environment, which is located in the volume V (t) at time t, if the integral relations governing the quasistatic electric field are localized via the generalized Gauss and Stokes theorems, the following relations are achieved. Details can be found in [26,27].

Gauss’ law:

(3) $$div\;\;D=\;0\;\;\;\;\;\;\;\;\;\;\mathrm{or}\;\;\;\;\;\;\;\;\;\;\;\;\;\;\;\;\;\;D_{i,\;i}=0\;\;\;\;\;\;\;\;\mathrm{in}\;\;\;\;V(t)$$

(4) $$\left[\left[D\right]\right]\;\cdot \;n\;=\;w_{f}\;\;\;\;\;\;\;\;\;\;\;\;\;\;\;\;\;\;\;\;\;\;\;\;\;\;\;\;\;\;\;\;\;\;\;\;\;\;\;\;\;\;\;\;\;\mathrm{on}\;\;\;\;\Gamma (t)$$

Faraday’s law:

(5) ∇×E=0      ⇒   E=−∇ϕ                           in V (t)

(6) $$n\times \left[\left[E\right]\right]=0\;\;\;\;\;\;\;\;\;\;\;\;\;\;\;\;\;\;\;\;\;\;\;\;\;\;\;\;\;\;\;\;\;\;\;\;\;\;\;\;\;\;\;\;\;\;\;\;\;\;\;\;\;\;\;\;\mathrm{on}\;\;\;\;\Gamma (t)$$

The expression below defines the constitutive relationship between the electric displacement, electric field, and polarization in a dielectric medium.

(7) $$D=\xi_{0}\;E+P\;\;\;\;\;\;\;\;\;\;\;\;\mathrm{or}\;\;\;\;\;\;\;\;D_{i}\equiv \xi_{0}E_{i}+P_{i}$$

Here, $w_{f}$ is the free surface charge distribution. $\left[\left[D\right]\right]\equiv D^{+}-D^{-}$ and $\left[\left[E\right]\right]\equiv E^{+}-E^{-}$ are jumps of D and E across the discontinuity surface [26].

### 2.3. Electro-Thermomechanical Balance Equations

Similarly, when the generalized Gauss and Stokes theorems are applied, if the global thermomechanical balance equations are localized, the following expressions are acquired:

Conservation of mass:

(8) $$\frac{d\rho }{dt}+\rho \;v_{i,i}=0\;\;\;\;\;\;\;\;\mathrm{or}\;\;\;\;\;\;\;\;\;\;\;\;\;\;\frac{\partial \rho }{\partial t}+{\left(\rho \;\;v_{i}\right)}_{,\;i}=0\;\;\;\;\mathrm{in}\;\;V(t)$$

(9) $$\left[\left[\;U\;\rho \;\right]\right]=0\;\;\;\;\;\;\;\;\;\;\;\;\;\;\;\;\;\;\;\;\;\;\;\;\;\;\;\;\;\;\;\;\;\;\;\;\;\;\;\;\;\;\;\;\;\;\;\;\;\;\;\;\;\;\;\;\;\;\;\;\;\;\;\;\;\;\;\;\;\;\;\;\mathrm{on}\;\;\;\;\Gamma (t)$$

In the present case, $\rho_{\;0}(X)$ is the known density of the media at the reference position, J (y,t) is the Jacobian, and the following relation can be stated:

(10) $$\rho_{\;0}\;(X)\;dV\;(X)=\rho \;\;(y,t)\;\;dv(y,t),\;\;\;\;\;\;\rho (y,t)=J^{-1}(y,t)\;\rho_{\;0}(X)$$

Linear momentum balance:

(11) $$\rho \;{\dot{v}}_{j}=\sigma_{ij,i}+B_{j}+P_{i}\;E_{j,\;i\;}\;\;\;\;\;\;\;\;\;\;\;\;\;\;\;\;\;\;\;\;\;\;\;\;\;\;\;\;\;\;\;\;\;\;\;\;\;\;\;\;\mathrm{in}\;V\;\;(t)$$

(12) $$\left[\left[n_{i}\sigma_{ij}+\rho \;v_{j}U\right]\right]=0\;\;\;\;\;\;\;\;\;\;\;\;\;\;\;\;\;\;\;\;\;\;\;\;\;\;\;\;\;\;\;\;\;\;\;\;\;\;\;\;\;\;\;\;\;\;\;\;\;\;\;\;\mathrm{on}\;\;\;\;\Gamma (t)$$

Angular momentum balance:

(13) $$\varepsilon_{kij}\;(\sigma_{ij}+P_{i}\;E_{j})=0\;\Rightarrow \;\;\;\;\;\;\;\;\varepsilon_{kij}\;{\bar{\sigma }}_{ij}=0\;\;\;\;\;\;\;\;\;\;\;\;\mathrm{in}\;\;\;\;V(t)$$

(14) $${\bar{\sigma }}_{ij}\equiv \sigma_{ij}+P_{i}\;E_{j}={\bar{\sigma }}_{ji}\;\;\;\;\mathrm{or}\;\;\;\;\;\;\;\;\sigma_{ij}={\bar{\sigma }}_{ij}-P_{i}\;E_{j}$$

(15) $$\varepsilon_{klj}x_{l}\left[\left[n_{i}\;\sigma_{ij}+\rho \;U\;v_{j}\right]\right]=0\;\;\;\;\;\;\;\;\;\;\;\;\;\;\;\;\;\;\;\;\;\;\;\;\;\;\;\;\;\;\;\;\mathrm{on}\;\;\;\;\Gamma (t)$$

Conservation of energy:

(16) $$\rho \;\dot{\varepsilon }\;\;\;=\;\sigma_{ij}\;v_{j,i}-q_{i,i}+\rho \;h+\rho \;h^{E}+\varepsilon_{kij}\;P_{i}\;E_{j}\;\omega_{k}\;\;\;\;\;\;\;\;\;\;\;\;\mathrm{in}\;\;V(t)$$

(17) $$\rho U\left[\left[\varepsilon +\frac{1}{2}{\left|v\right|}^{2}\right]\right]+n_{i}\;[[\sigma_{ij}\;v_{j}-q_{i}]]=0\;\;\;\;\;\;\;\;\;\;\;\;\;\;\;\;\mathrm{on}\;\;\;\;\Gamma (t)$$

Second law of thermodynamics:

(18) $$\rho \;\dot{\eta }-\frac{1}{\theta }\;\rho \;h+\frac{1}{\theta }\;\;q_{i,i}-\frac{1}{\theta^{\;2}}\;\;q_{i}\;\theta_{i}\geq 0\;\;\;\;\;\;\;\;\;\;\;\;\;\;\;\;\;\;\;\;\;\;\;\;\;\;\;\;\mathrm{in}\;\;V(t)$$

(19) $$\rho \;U\left[\left[\eta \right]\right]-\left[\left[\frac{n\cdot q}{\theta }\right]\right]\leq 0\;\;\;\;\;\;\;\;\;\;\;\;\;\;\;\;\;\;\;\;\;\;\;\;\;\;\;\;\;\;\;\;\;\;\;\;\;\;\;\;\;\;\;\;\;\;\;\;\;\;\;\;\mathrm{on}\;\;\;\;\Gamma (t)$$

In these expressions, the physical interpretations of several symbols are as follows: U is the relative displacement velocity of the impermanent surface according to the media ($U\equiv u_{n}-v_{n}=(u-v)\cdot n$), ${\bar{\sigma }}_{ij}^{E}$ is the polarization stress tensor (${\bar{\sigma }}_{ij}^{E}=P_{i}\;E_{j}$), Π is the polarization vector per unit of mass ($\Pi \equiv \frac{P}{\rho }$), *h* is the energy source per unit mass, *h^E^* is the electrostatic energy source ($\rho \;h^{E}=E_{i}\;\overset{*}{P_{i}}+{\bar{\sigma }}_{ij}^{E}\;\;d_{ji}$), and $\omega_{k}$ is the angular velocity ($\omega =\frac{1}{2}\;\nabla \times v$) [24,25]. Using these expressions, the localized linear momentum and energy equations given expressions (11) and (16), are obtained as follows:

(20) $$\rho \;{\dot{v}}_{j}=B_{j}+{\bar{\sigma }}_{ij,i}-P_{i,i}\;E_{j}\;\;\;\;\;\;\;\;\;\;\;\;\;\;\;\;\;\;\;\;\;\;\;\;\;\;\;\;\;\;\;\mathrm{in}\;\;V(t)$$

(21) $$\rho \;\;\dot{\varepsilon }=\sigma_{ij}\;v_{j,i}-q_{i,i}+\rho \;h+\rho \;E\cdot \dot{\Pi }\;\;\;\;\;\;\;\;\;\;\;\;\;\;\;\;\mathrm{in}\;\;V(t)$$

## 3. Thermodynamic Conditions and Constitution Model

If (ρ h ) is obtained from the local energy Equation (21) and substituted into the entropy inequality (18), the following inequality is obtained:

(22) $$\frac{\rho }{\theta }\;(\dot{\varepsilon }-\theta \;\dot{\eta }-E\cdot \dot{\Pi })+\frac{1}{\theta }\sigma_{ij}\;v_{j,i}-\frac{1}{\theta^{2}}\;q_{i}\;\theta_{i}\geq 0$$

Since the material derivatives of entropy density and polarization in this expression cannot be controlled within a thermodynamic process, a Legendre transformation, defined as follows, can be used to transform the derivatives of these quantities into controllable quantities θ and E.

(23) ψ≡ε−θη−E⋅Π=ε−θη−ρ  Ei1Pi

Since θ is a positive value, multiplying it by Inequality (22) will not change the inequality. The term $v_{j,i}$ in Inequality (22) is defined as $v_{j,i}=d_{ji}+w_{ji}$ and is known as the velocity gradient tensor [25]. Here, $d_{ji}$ is the strain rate tensor and is symmetric, while $w_{ji}$ is the spin tensor and is antisymmetric. The stress tensor in Inequality (22) is derived from mechanical and electrical loads and is not symmetric. If the stress expression given by Expression (14) is written in place of this stress tensor, the resulting new entropy inequality will contain only a symmetric-stress tensor, thus allowing us to benefit from the advantages of a symmetric tensor. Consequently, the entropy inequality in terms of new terms is written as follows:

(24) $$-\rho \;(\dot{\psi }+\dot{\theta }\;\eta )+{\bar{\sigma }}_{ij}\;d_{ji}-P_{i}\;E_{j}\;v_{j,i}-\frac{1}{\theta }\;q_{i}\;\theta_{i}-P_{i}\;{\dot{E}}_{i}\geq \;\;\;\;0$$

If the material derivative of the Green deformation tensor, defined as $C_{IJ}\;(X,\;t)=y_{j,I}\;y_{j,J}$, is taken, it is written as ${\dot{C}}_{IJ}=2\;d_{ij}y_{i,I}y_{j,J}$ in terms of $d_{ij}$. When the Expression (10) for ρ are substituted in Inequality (24), and we multiply the inequality by Jacobian (J), the following inequality is obtained:

(25) $$-\rho_{0}\;(\dot{\psi }+\dot{\theta }\;\eta )+\frac{1}{2}\;J\;{\bar{\sigma }}_{ij}\;{\dot{C}}_{IJ}X_{I,i}X_{J,j}-J\;P_{i}\;E_{j}\;v_{j,i}-\frac{1}{\theta }\;J\;q_{i}\;\theta_{i}-J\;P_{i}\;{\dot{E}}_{i}\geq 0$$

The stress potential (Σ) associated with ψ can be defined as follows:

(26) $$\Sigma \equiv \rho_{0}\;\;\psi$$

Σ will henceforth be referred to as free energy. By substituting the free energy given by the definition in (26) into (25), the inequality takes the following new form:

(27) $$-(\dot{\Sigma }+\;\rho_{0}\;\eta \;\dot{\theta })+\frac{1}{2}\;J\;{\bar{\sigma }}_{ij}\;{\dot{C}}_{IJ}X_{I,i}X_{J,j}-J\;P_{i}\;E_{j}\;v_{j,i}-\frac{1}{\theta }\;J\;q_{i}\;\theta_{i}-J\;P_{i}\;{\dot{E}}_{i}\geq 0$$

When this inequality is put into a material form, the following inequality emerges:

(28) $$-(\dot{\Sigma }+\rho_{0}\;\eta \;\;\dot{\theta }\;)+\frac{1}{2}\;{\bar{\sigma }}_{IJ}\;{\dot{C}}_{IJ}-\frac{1}{\theta }\;\;\theta_{I}\;Q_{I}-\Pi_{I}\;{\dot{E}}_{I}\;\;\geq 0$$

The relations between the material and spatial components of the magnitudes that appear in Inequality (28) are expressed below [24,25]:

(29) $${\dot{C}}_{IJ}=2\;\;d_{ij}\;y_{i,I}\;y_{j,J}\;\;\;\;\Rightarrow \;\;\;\;d_{ij}=\frac{1}{2}{\dot{C}}_{IJ}X_{I,i}X_{J,j}$$

(30) $${\bar{\sigma }}_{IJ}\equiv J\;X_{I,i}\;X_{J,j}\;{\bar{\sigma }}_{ij}\;\;\;\;\Rightarrow \;\;\;\;{\bar{\sigma }}_{ij}=J^{-1}\;y_{i,I}\;y_{j,J}\;{\bar{\sigma }}_{IJ}$$

(31) $$Q_{I}\equiv J\;X_{I,i}\;q_{i}\;\;\;\;\;\;\Rightarrow \;\;\;\;\;\;q_{i}=J^{-1}\;y_{i,I}\;Q_{I}$$

(32) $$\Pi_{I}\equiv J\;X_{I,i}\;P_{i}=\rho_{0}\;X_{I,i}\;\frac{P_{i}}{\rho }=\rho_{0}\;X_{I,i}\;\Pi_{i}\;\;\;\;\Rightarrow \;\;P_{i}=J^{-1}\;y_{i,I}\;\Pi_{I}$$

(33) $$E_{I}\equiv y_{i,I}\;E_{i}\;\;\;\;\Rightarrow \;\;E_{i}=X_{I,i}\;E_{I}$$

(34) $$\theta_{I}\equiv y_{i,I}\;\theta_{i}\Rightarrow \theta_{i}=X_{I,i}\;\theta_{I}$$

Inequality (28) is a general expression of entropy production for the thermomechanical media that are under the influence of a quasi-electrostatic field and have elastic properties. In order to use this inequality, it is necessary to know how the thermodynamic potential (Σ) depends on independent variables, and which variables these are. Accordingly, Σ’s arguments can be put forward by selecting a specific material. In this study, electroelastic material is considered. In accordance with the selection of this material, the Σ arguments—and the variables they depend on—, are obtained via constitutive axioms [24,25]. The stress potential (Σ) of material point X at time t in the material considered, is assumed to depend on the motion and temperature history—and the electric field history—of all the material points constituting the object. By drawing on the implications of the axioms of causality, determinism, objectivity, proximity, memory, and consistency, the arguments to which Σ is dependent in piezoelectric and elastic media subjected to an electromechanical load without heat conduction can be put forward as follows:

(35) $$\Sigma \;(X,t)=\Sigma \;\;[C_{IJ}(X,t)\;,\;\;E_{I}(X,t),\;\theta \;(X,t)]$$

Assuming that the material is homogeneous, X is removed from the arguments from which the expression given in (35) is related. If the material derivative of Expression (35) is taken and substituted into Inequality (28), and if the necessary operations are performed, the formulations of the constitutive equations for the piezoelectric–elastic material under consideration can be summarized as follows:

(36) $${\bar{\sigma }}_{IJ}=2\;\frac{\partial \;\Sigma }{\partial \;C_{IJ}}$$

(37) $$\Pi_{I}=-\frac{\partial \;\Sigma }{\partial \;E_{I}}$$

(38) $$\eta =-\frac{1}{\rho_{0}}\;\;\frac{\partial \;\Sigma }{\partial \;\theta }$$

(39) $$Q_{I}=0$$

There is a relationship in the form of $C_{IJ}=2\;E_{IJ}+\delta_{IJ}$ between the Green deformation tensor and the strain tensor. In linear theory it is in the form of $E_{IJ}≅{\tilde{E}}_{IJ}\equiv \;\frac{1}{2}\;\;(U_{I,J}+U_{J,I})$. Therefore, the arguments of the stress potential given by Relation (35) and Equation (36) can be written as follows:

(40) $$\Sigma =\Sigma \;({\tilde{E}}_{IJ},E_{I},\;\theta )$$

(41) $$\;{\bar{\sigma }}_{IJ}=\frac{\partial \;\Sigma }{\partial \;\;{\tilde{E}}_{IJ}}$$

From the equations given with Expressions (37) and (41), the polarization and the symmetric stress are obtained from Σ. Therefore, the clear form of Σ, which takes place as a constitutive function with certain arguments, is necessary to determine. In this study, the material is assumed to be a general anisotropic medium. In this context, the elastic behavior and polarization response of the media are defined by expanding the stress potential function into a power series based on the components of its dependent variables. All the interactions are acknowledged to be linear in this study. In addition, the first-order interaction of the strain tensor with the electric field vector is also considered. These assumptions have been considered in operations related to the following parts.

## 4. Determination of the Constitutive Equations and Field Equations

The ${\tilde{E}}_{PR}$ strain tensor and $E_{P}$ electric field vector change via coordinate transformations because they depend on the material coordinates. Since θ is a scalar quantity, it is not affected by coordinate transformations. Therefore, for ease of notation, the dependence of the stress potential on temperature will not be shown. Once the stress potential function (Σ), whose arguments are presented in Expression (40), is accepted analytically in terms of ${\tilde{E}}_{PR}$ and $E_{P}$, and is expanded to a Taylor series around ${\tilde{E}}_{PR}=\;0$ and $E_{P}=\;0$, the stress potential function (Σ) is written as follows:

(42) $$\Sigma \;({\tilde{E}}_{PR},\;E_{P})\;\;=\;\Sigma_{0}+\;\Sigma_{PR}\;{\tilde{E}}_{PR}+\;\beta_{P}\;E_{P}\;+\;\frac{1}{2}\Sigma_{PRKL}{\tilde{E}}_{PR}{\tilde{E}}_{KL}+\frac{1}{2}\;\beta_{PL}E_{P}E_{L}\;+\mu_{PRL}{\tilde{E}}_{PR}E_{L}+\ldots$$

The coefficients in Expression (42) depend only on temperature and are defined as follows:

(43) $$\Sigma_{0}\equiv \Sigma (\underline{\underline{0}}\;,\;\underline{0}),\Sigma_{PR}\equiv {\left.\frac{\partial \Sigma }{\partial {\tilde{E}}_{PR}}\right|}_{0},\beta_{P}\equiv {\left.\frac{\partial \Sigma }{\partial E_{P}}\right|}_{0},\Sigma_{PRKL}\equiv {\left.\frac{\partial^{2}\Sigma }{\partial {\tilde{E}}_{PR}\;\partial \;{\tilde{E}}_{KL}}\right|}_{0},\beta_{PL}\equiv {\left.\frac{\partial^{2}\Sigma }{\partial \;E_{P}\;\partial \;E_{L}}\right|}_{0},\mu_{PRL}\equiv \frac{1}{2}{\left.\frac{\partial^{2}\Sigma }{\partial {\tilde{E}}_{PR}\;\partial \;E_{L\;}}\right|}_{0}$$

Because of the symmetry of the strain tensor ${\tilde{E}}_{PR}$, and because the derivatives in the definitions in (43) do not depend on the order, these coefficients have the following symmetry properties:

(44) $$\Sigma_{PR}=\Sigma_{RP},\;\;\;\;\;\;\;\;\Sigma_{PRKL}=\Sigma_{RPKL}=\Sigma_{PRLK}=\Sigma_{KLPR},\;\;\;\;\;\;\;\;\;\;\beta_{PL}=\;\beta_{LP},\;\;\;\;\;\;\;\;\;\;\mu_{PRL}=\;\mu_{RPL}$$

Since mechanical interactions are considered linear in this study, the linear theory in continuum mechanics can be expressed as follows [25,28]:

$$y_{i,I}=\lambda_{iI}+u_{i,I},\;\;\;\;\;\;X_{I,i}=\Lambda_{Ii}-U_{I,i},\;\;\;\;\;\;\;\;y_{i,I}y_{j,J}=\lambda_{iI}\;\lambda_{jJ},\;\;\;\;\;\;\;\;X_{I,i}X_{J,j}=\lambda_{iI}\;\lambda_{jJ},\;\;\;\;\;\;\;\;\;\;\;{\tilde{Ε}}_{IJ}\equiv \;\frac{1}{2}\;(\;U_{I,J}+U_{J,I}),\;\;{\tilde{Ε}}_{IJ}≅\lambda_{iI}\;\lambda_{jJ}\;{\tilde{e}}_{ij},\;\;\;\;\;\;\;\;\in_{ij}\;≅\lambda_{iI}\;\lambda_{jJ}\;{\tilde{Ε}}_{IJ},\;\;\;\;\;\;\;\;{\tilde{e}}_{ij}\;=\;\in_{ij}\;\equiv \;\frac{1}{2}\;(u_{i,j}+u_{j,i}),\;\;\;\;\;\;\;\;\;{\tilde{Ε}}_{IJ}=\frac{1}{2}\;\lambda_{iI}\;\lambda_{jJ}\;(u_{i,j}+u_{j,i})\;,\;\;d_{mn}=\frac{\partial \;\;{\tilde{Ε}}_{MN}}{\partial \;t}\;X_{M,m}\;X_{N,n}=\frac{\partial \in_{mn}}{\partial \;t}\;,\;\;\;\;\;\;\;\;\;\;d_{mn}=\frac{\partial \;(u_{m,n})}{\partial \;t}=\frac{1}{2}\;({\dot{u}}_{m,n}+{\dot{u}}_{n,m}),\;\;\;\;\;\;\;\;\;\dot{\varepsilon }\approx \frac{\partial \;\varepsilon }{\partial \;t},$$

(45) $${\dot{v}}_{m}\;≅\;\;\frac{\partial^{2}u_{m}}{\partial \;t^{2}},\;\;\;\;\;\;\;\;J^{-1}≅1-u_{m,m},\;\;\;\;\rho ≅\rho_{0}\;(1-u_{m,m}),\;\;\;\;\;\;y_{m,M}\;y_{n,N}\;E_{I}\equiv \lambda_{mM}\;\lambda_{nN}\;\lambda_{iI}\;E_{i}$$

Expressions giving the spatial form of symmetric stress and polarization can be written in linear theory as follows:

(46) $${\bar{\sigma }}_{ij}=(1-u_{k,k})\;\frac{\partial \;\Sigma }{\partial \in_{ij}}$$

(47) $$P_{i}=-(1-u_{k,k})\;\;\frac{\partial \;\Sigma }{\partial \;E_{i}}$$

In linear theory, the dependent arguments of the stress potential function (Σ) are written in the spatial form as follows:

(48) $$\Sigma =\Sigma \;(\in_{pr},\;E_{p})$$

When this function is adopted from the points $\in_{pr}$ and $E_{p}$, if it expands in the Taylor series around $\in_{pr}\;=\;0$ and $E_{p}=0$, the following expression is obtained:

(49) $$\Sigma \;(\in_{pr},E_{p})=\Sigma_{0}+\Sigma_{pr}\in_{pr}+\beta_{p}E_{p}+\frac{1}{2}\Sigma_{prkl}\;\in_{pr}\in_{kl}+\frac{1}{2}\;\beta_{pl}E_{p}\;E_{l}+\mu_{prl}\in_{pr}E_{l}+\ldots$$

The spatial material tensors $\Sigma_{pr}$, $\Sigma_{prkl}$, $\beta_{pl}$, and $\mu_{prl}$ in this equation have identical symmetry features as the tensors of $\;\Sigma_{PR}$, $\Sigma_{PRKL}$, $\beta_{PL}$, and $\mu_{PRL}$ given in Expression (44), and are described as follows:

(50) $$\Sigma_{pr}\equiv \lambda_{pP}\;\lambda_{rR}\;\Sigma_{PR},\;\;\;\;\Sigma_{prkl}\equiv \lambda_{pP}\;\lambda_{rR}\;\lambda_{kK}\;\lambda_{lL}\;\Sigma_{PRKL},\;\;\;\;\;\;\beta_{pl}\equiv \lambda_{pP}\;\lambda_{lL}\;\beta_{PL},\;\;\;\;\;\;\;\;\mu_{prl}\equiv \lambda_{pP}\;\lambda_{rR}\;\lambda_{lL}\;\mu_{PRL}$$

If the derivatives in Expressions (46) and (47) are taken from the Expression (49) and replaced, the following expressions are obtained:

(51) $${\bar{\sigma }}_{ij}=(1-u_{k,k})\;(\Sigma_{ij}+\Sigma_{ijkl}\;\in_{kl}\;+\;\mu_{ijl}\;E_{l})$$

(52) $$P_{i}=-(1-u_{k,k})\;(\beta_{i}+\beta_{il}\;E_{l}+\mu_{kli}\in_{kl})$$

Under natural conditions, which are described with $\in_{pr}\;=\;0$, $E_{p}=0$, if stress and polarization is being zero considered, $\Sigma_{ij}=0$ and $\beta_{i}=0$ can be taken. The Relationships (51) and (52) are written as follows in terms of the linear constituents of the displacement gradient and due to the symmetries $\Sigma_{ijkl}=\Sigma_{ijlk}$ and $\mu_{kli}=\mu_{lki}$ of the coefficients $\Sigma_{ijkl}$ and $\mu_{kli}$. Here, the linear terms of both the mechanical and electrical interactions are taken into account:

(53) $${\bar{\sigma }}_{ij}=\Sigma_{ijkl}\;u_{k,l}+\mu_{ijl}\;E_{l}$$

(54) $$P_{i}=-(\beta_{il}\;E_{l}+\mu_{kli}\;u_{k,l})$$

Writing the Expressions (53) and (54) into Equation (14), and considering the linear terms of the strain tensor and the electric field vector, the constitutive equation of asymmetric stress is found in the spatial form given below:

(55) $$\sigma_{ij}=\;\Sigma_{ijkl}\;u_{k,l}\;+\mu_{ijl}\;E_{l}$$

Considering the linear terms of the strain tensor and the electric field vector, the symmetric stress and the asymmetric stress are obtained in the same form. In the spatial coordinates the constitutive equations of the symmetric stress, the polarization field, and the asymmetric stress have been obtained with Equations (53)–(55) in terms of the displacement gradient and electric field vector. To obtain the field equations, these constitutive relations are written into the balance expressions. Writing Expression (54) into Equation (7) and applying Expression (5), the total electric displacement vector is obtained as follows:

(56) $$D_{i}=-\Omega_{il}\;\phi_{l}\;-\mu_{kli}\;u_{k,l}$$

The $\Omega_{il}$ coefficient in Equation (56) is described as $\Omega_{il}\equiv \;\xi_{0}\;\delta_{il}-\beta_{il}$. Considering the acceptances made in this study, taking the divergence of Equation (56) and substituting it into Expression (3) yields the following equation:

(57) $$0=D_{i,i}=-\Omega_{il}\;\phi_{l,i}-\mu_{kli}\;u_{k,li}$$

Writing Expression (5) in the constitutive equations given with Expressions (53) and (54), and considering the acceptances made in this study, taking the divergences of these equations and then substituting them into Equation (20) yields the following field equation:

(58) $$\rho_{0}\frac{\partial^{2}u_{j}}{\partial t^{2}}=\rho_{0}\;B_{j}+\Sigma_{ijkl}\;u_{k,li}-\mu_{ijl}\;\phi_{l,i}$$

Expressions (57) and (58) are field equations and these equations include the $u_{k}$ and ϕ unknowns. The solving of these expressions under the starting and frontier conditions given in accordance with the problem forms the mathematical formulation of the boundary value problem that must be considered. Finally, the system consisting of Expressions (53) and (54), together with the boundary conditions in the Bouncing Conditions (4), (12), and (6), creates the governing equations of the frontier worth problems associated with the media considered in this study. If the mentioned boundary conditions are written explicitly, it can be easily shown that they are of the form $D_{n}=D_{n}^{+}-\omega_{f}$, $n_{i}\;\sigma_{ij}=\sigma_{j}^{+}$, and $E_{k}=E_{k}^{+}$.

### For Static Analysis Simplified Governing Equations

The Static testing of piezoceramic actuators has received limited attention compared to their dynamic applications. However, deeper understanding of the static behavior of piezoactuators is essential for their effective utilization in structural applications [29]. Crawley and Anderson [30] used ADINA to model piezoceramic—driven structures, performing static deflection tests to validate their solutions in ADINA. In the ADINA Validation Guide, they stated that the strain results between the experimental results and FE results were similar. Crawley and Lazarus [31] conducted plate experiments that demonstrated significant agreement with analytically predicted deformations, confirming the dependence of the piezoelectric mechanical/electrical coupling coefficient on the induced voltage. To validate the results of the analytical structural modeling of double composite beams with spatially distributed, induced strain actuators, Chandra and Chopra [32] also conducted static deflection experiments.

The analytical formulations carried out by Crawley and de Luis [33] are considered the starting point for much of the subsequent work, given that they provided the starting point for the development of smart structures modeling using both surface-mounted and beam-embedded piezoactuators.

By ignoring the time derivative terms in Equations (11) and (21), the general governing relations are shortened for the static solution. Moreover, during deformation, assuming the absence of any heat generation, the relevant governing electroelastic relations can be expressed as follows:

(59) $$\sigma_{ij,i}+B_{j}+P_{i}\;E_{j,i}=0,\;\;\;\;\;\;\;\;j=1,2,3$$

(60) $$D_{i,i}=0$$

(61) $$q_{i,i}=0$$

Although the thermodynamic formulation admits asymmetric stress due to polarization–field couples, the commercial FEM elements used here (PLANE13/PLANE182) implement the classical linear piezoelectric constitutive law with symmetric Cauchy stress. In this study, we specialize our general relations by (i) neglecting body couples; (ii) adopting small-strain, compressible elasticity; and (iii) retaining only first-order electro-mechanical coupling. Under these assumptions, the constitutive equations are reduced to $\sigma_{ij}=\;\Sigma_{ijkl}\;u_{k,l}\;+\mu_{ijl}\;E_{l}$ and $D_{i}=-\Omega_{il}\;\phi_{l}-\mu_{kli}\;u_{k,l}$. Therefore, additional asymmetric terms are not used in our numerical implementation; a user-defined element would be required to explore them, which we leave for future work.

## 5. Numerical Solution of a Smart Structure for Static Analysis

The modeling and analysis of piezoelectric smart structures can be performed via finite element software packages. This approach facilitates the efficient simulation of smart structures, and the underlying mathematical models are typically formulated via the finite element method [34,35,36,37,38,39,40]. Software tools such as ANSYS, ABAQUS, and MSC/NASTRAN are commonly employed to derive and analyze these mathematical models. Numerical analysis and FEM solutions (especially ANSYS) provide an important means of understanding the electromechanical behaviors of smart materials. In this study, numerical analyses are performed via the finite element method ANSYS (APDL) software (2024 R1). The study presented in [41] investigated cantilever smart structures composed of aluminum beams integrated with lead–zirconate–titanate (PZT) patches. Numerical, via FEM solution (ANSYS), and experimental analyses were conducted in parallel for two distinct cases. In the first case, a smart structure with a single PZT patch was employed to control free vibrations induced by initial tip displacement. In the second case, a smart structure with two PZT patches was utilized for forced vibration control under harmonic excitation.

### 5.1. General Principles for Modelling of a Smart Structure

**Figure 2:**
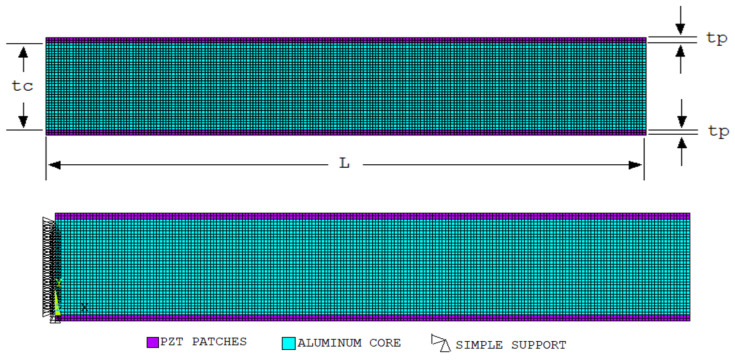
Geometry of the two-dimensional beam–patch model used in the FEM analysis.

The smart structure shown in Figure 2 is considered as follows. For smart structures, the global coordinate system aligns the x-axis with the lengthwise direction of said structure; the y-axis is assigned to the depth direction, and the z-axis is used as the through thickness direction. The smart structures comprise a metallic core with piezo patches on its surface, as illustrated in Figure 2. PZT-5H is selected as the material for the piezo patches, which is transversely isotropic, inherently.

### 5.2. Two-Dimensional Finite Element Model

In this section, a two-dimensional finite element model of a beam–patch system, including the selected element types, boundary conditions, and mesh convergence studies, is presented. All two-dimensional analyses were conducted under small-strain, isothermal, and electrostatic assumptions.

#### 5.2.1. Element Types and Assumptions

- ANSYS PLANE182 (two-dimensional structural, four-node, linear) has been used as the element type for the beam;
- ANSYS PLANE13 (two-dimensional piezoelectric conjugate area element, four nodal points) has been used as the element type for PZT patches;
- Analyses have been carried out according to the plane-strain assumption, and the polarization direction of the PZT patches has been defined in the direction normal to the two-dimensional plane (+y). Thus, the electric field generated by the applied voltage in the 2D model produces a longitudinal-bending response via piezo-coupling terms.
- It is assumed that the interfaces of the beam and PZT patches are perfectly bonded.

PZT-5H has been selected for the piezoelectric patches, and aluminum has been selected as the beam material. The material properties of both PZT-5H and aluminum are presented in Table 1.

#### 5.2.2. Geometry, Boundary Conditions, and Polarization Definition

The geometry of the two-dimensional model is presented in Figure 2. PZT patches have been bonded to the top and bottom of an aluminum beam. The thickness of the PZT patches (t_p_) is 0.001 m, the thickness of the aluminum core (t_c_) is 0.016 m, and the length (*L*) of the aluminum beam with the PZT patches is 0.1 m.

The aluminum beam is supported at its left edge ($u_{x}=u_{y}=0$ for all nodes in the aluminum beam along the left end). The vertical end displacement w(L) is used as the reaction quantity at the free end.

**Figure 3:**
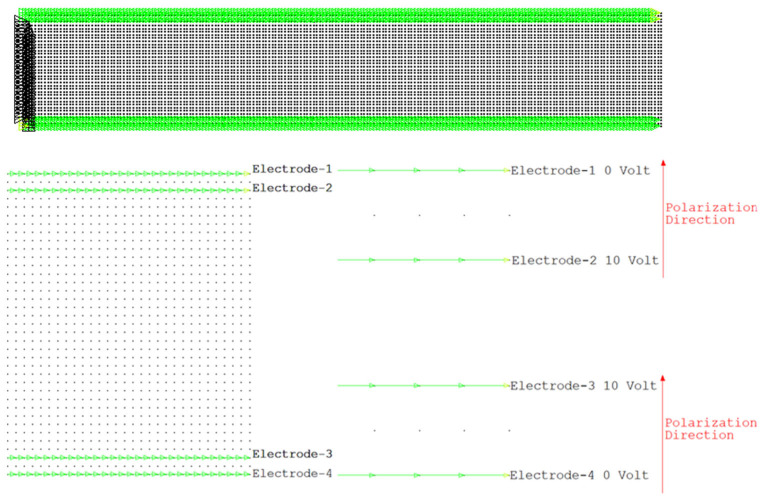
Electrodes and polarization direction in PZT.

The patches are then attached to the upper and lower surfaces of the beam. Both patches are polarized in the (+y) direction. In order to produce bending, opposite potentials are applied to the upper and lower patches. The potentials are applied to the upper and lower patches in opposite directions to produce bending. Ten volts are applied to the electrodes at the adhesion interface, and the upper electrode of the upper patch and the lower electrode of the lower patch are assigned as the reference (ground). When the upper and lower patches are operated in opposition, a pure bending moment occurs. The polarization directions of the PZT patches and the associated electrode configuration have been shown in Figure 3.

#### 5.2.3. Mesh and Convergence Study

The mesh structure of the two-dimensional model is configured to be compatible with the beam–patch interface. The mesh structure of the two-dimensional beam–model has been examined by selecting different element sizes. For element length (h), $h_{1}=5\;\times \;10^{-3}\;m,\;$$h_{2}=25\;\times \;10^{-4}\;m,\;$$h_{3}=1\;\times \;10^{-3}\;m,\;$$h_{4}=5\;\times \;10^{-4}\;m,\;$$h_{5}=25\;\times \;10^{-5}\;m,\;$$h_{6}=1\;\times \;10^{-4}\;m,$ and $h_{7}=5\;\times \;10^{-5}\;m$ values have been taken. The free-end displacement w(L;h) has been calculated for each element size and the successive difference has been reported as the following expression:

(62) $$r\;(h_{i})\;=\;\frac{\left|\;w(L;h_{i})-w(L;h_{i-1})\;\right|}{\left|\;w(L;h_{i-1})\;\right|}$$

The target for the convergence criterion is to have an r less than 1% at two consecutive levels. As the element size decreases, the desired convergence criterion has been achieved. The results have been presented in Table 2.

**Figure 4:**
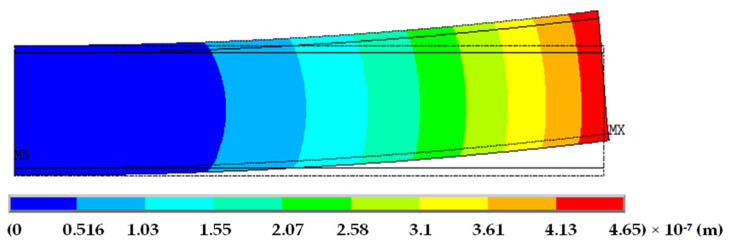
Tip displacement *w*(*L*) of the 2D beam-patch model for 10 V loading and element size *h* = 5 × 10^−4^ m.

The end displacement *w*(*L*) of the 2D beam-patch model for a 10 V load and element size *h* = 5 × 10^−4^ m, which is one of the 2D ANSYS solutions, is given in Figure 4.

#### 5.2.4. Analytical Solution

The equivalent bending moment due to the electrical stresses applied to the PZT patches has been derived with a closed–form analytical model based on the Euler–Bernoulli beam theory summarized below. The bending stiffness per unit width (YI in Euler–Bernoulli) has been obtained as follows:

(63) $$YI\;\;=\;Y_{c}\;\frac{t_{c}^{3}}{12}+\;2\;Y_{p}\;(\;\frac{t_{p}^{3}}{12}\;+\;t_{p}\;z_{t}^{2}\;)$$

Here, Y is the Young (elasticity) modulus, I  is the moment of inertia of the section, $t_{c}$ is the aluminum core thickness, $t_{p}$ is the PZT patch thickness, $z_{t}$ is $z_{t}=\frac{t_{c}+t_{p}}{2}$, and $z_{b}$ is $z_{b}=-\frac{t_{c}+t_{p}}{2}$. When the upper and lower PZT patches are operated in opposition, the axial force cancels and a pure bending moment occurs on the beam:

(64) $$M^{*}=Y_{p}\;\alpha_{act}\;t_{p}\;(z_{t}-z_{b})\;\;\;\;\;\;\;\;\mathrm{or}\;\;\;\;\;\;\;\;\;\;\;\;M^{*}=Y_{p}\;d_{31}\;(\;t_{c}+t_{p})$$

Here, $\alpha_{act}$ is $\alpha_{act}=d_{31}\;\frac{V}{t_{p}}$, $d_{31}$ is the piezoelectric coefficient, and V is the applied electric potential. The curvature ($K_{0}$) is $K_{0}=\frac{M^{*}}{Y\;I}$ and the displacement (w) in the absence of an external load is $w\prime \prime (x)=K_{0}\Rightarrow w\;(x)=\frac{K_{0}}{2}\;x^{2}$ for w (0)=w′(0)=0. The tip displacement (x=L) has been written as follows:

(65) $$w(L)=w_{tip}=\frac{K_{0}\;L^{2}}{2}=\frac{M^{*}\;L^{2}}{2\;\;Y\;I}$$

In this study, the following numerical values have been adopted for the analytical solution: the elasticity modulus of the aluminum core $Y_{c}=70\;\mathrm{GPa},$ and the elasticity modulus of PZT-5H $Y_{p}=63\;\mathrm{GPa},$ with piezoelectric coefficient $d_{31}=274\;\mathrm{pm}/V$. When a voltage of V = 10 V is applied to the PZT patches, the free-end displacement *w*(*L*) is computed analytically using Equation (65) for the given material and geometric parameters. The resulting analytical value is quantitatively compared with the results from ANSYS obtained at each mesh level of the convergence study. This comparison is presented in Table 3.

Table 3 shows that the ANSYS tip displacement converges monotonically toward a stable value as the element size is reduced. The successive difference ratio r, defined by Equation (62), falls below 1% at *h* = 2.5 × 10^−4^ m and reaches 0% at *h* = 5 × 10^−5^ m, confirming that full convergence is achieved at *h* = 1 × 10^−4^ m in accordance with the adopted criterion of r < 1% at two consecutive levels. The converged ANSYS value, *w*(*L*) = 4.71 × 10^−7^ m, exceeds the analytical Euler–Bernoulli result of 4.45 × 10^−7^ m by 5.8%. This discrepancy is physically expected and does not indicate a modeling error. The 2D finite element model is formulated under the plane-strain assumption, which introduces an additional lateral stiffening constraint absent in the one-dimensional Euler–Bernoulli formulation, while the beam theory itself neglects the shear deformation that the 2D elasticity model partially captures through its two-dimensional stress state. Given these inherent differences between the two formulations, the observed level of agreement observed is considered satisfactory for validating the global electromechanical response of the beam–patch system.

### 5.3. Three-Dimensional Finite Element Model

This section presents a 3D electroelastic model of a smart cantilever beam with surface-bonded piezoelectric patches, and the influence of 3D element type selection on predicted tip deflection and interface stresses is examined. In a baseline configuration with full-length patches bonded to the top and bottom surfaces, interfacial shear and peel stresses at the patch–core interface are evaluated in terms of delamination risk. All analyses have been carried out under small-strain, isothermal, and electrostatic assumptions. PZT-5H has been chosen for the piezoelectric patches, and aluminum has been chosen as the beam material. The material properties of both PZT-5H and aluminum have previously been presented in Table 1.

**Figure 5:**
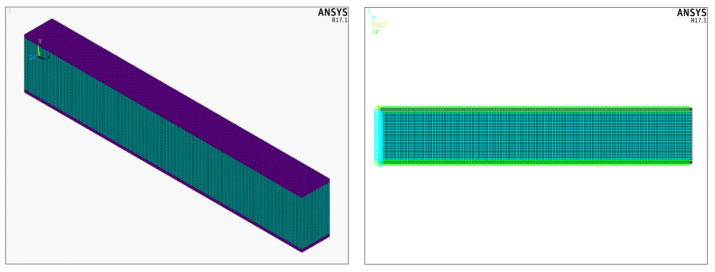
Modeling with ANSYS of a smart cantilever structure, and 10 V loading to the piezoelectric patches.

The geometry of the three-dimensional model is shown in Figure 5. The aluminum beam has a length of *L* = 0.10 m, a width of *b* = 0.01 m, and a thickness of *t_c_* = 0.016 m. The PZT-5H patches are bonded to the top and bottom surfaces of the beam and, in the baseline configuration, extend over the full beam length L, with a thickness of *t_p_* = 0.001 m and the same width *b* as the beam. The aluminum beam is clamped at its left end by constraining all translational degrees of freedom of the nodes on this edge, while the vertical displacement of the free end *w*(*L*) is taken as the performance criterion. Perfect bonding is assumed at the beam–patch interfaces; the adhesive layer is not modeled explicitly, and the computed interface shear and peel stresses are therefore used as indicators of delamination risk.

In the 3D model, a uniform element size of *h* = 5 × 10^−4^ m is used in all cases. This mesh size has been chosen because it provides an analytical solution and ANSYS result accuracy for the tip displacement in the 2D convergence study and is sufficient around the patch ends and the fixed support to resolve the interface stress gradients, which are the main focus of the 3D analyses. As in the 2D model, PZT-5H patches are bonded to the top and bottom surfaces of the beam, and both patches are polarized in the +*y* direction. An electric potential of 10 V is applied to the electrodes at the patch–beam interfaces, while the outer electrodes (top of the upper patch and bottom of the lower patch) are grounded. When the top and bottom patches are driven in opposite directions, a nearly pure bending moment is generated.

In the configuration where PZT patches are bonded along the full length of the top and bottom surfaces of the beam, two different element type combinations are considered for the beam and the patches. In the first case, the aluminum beam is modeled with SOLID185 and the PZT patches are modeled with SOLID5. These are eight-node, first-order brick elements (SOLID185: structural, linear elasticity; and SOLID5: coupled-field with DOF UX, UY, UZ, and VOLT). Although this combination leads to relatively fast solutions, bending deformation and interface stress gradients are captured more coarsely due to the lower-order interpolation, which results in a “softening” of peak stresses near the patch ends and at the fixed support. In the second case, the beam is modeled with SOLID186 and the PZT patches are modeled with SOLID226. These are 20-node, second-order brick elements (SOLID186: structural; and SOLID226: coupled-field with DOF UX, UY, UZ, and VOLT). The higher-order shape functions provide a much more accurate representation of bending and through-thickness stress gradients in the end regions, leading to a more reliable estimation of interface peak stresses and faster convergence of the free-end displacement *w*(*L*). The main drawback of this combination is the increased computational cost due to the larger number of nodes and degrees of freedom per element.

**Figure 6:**
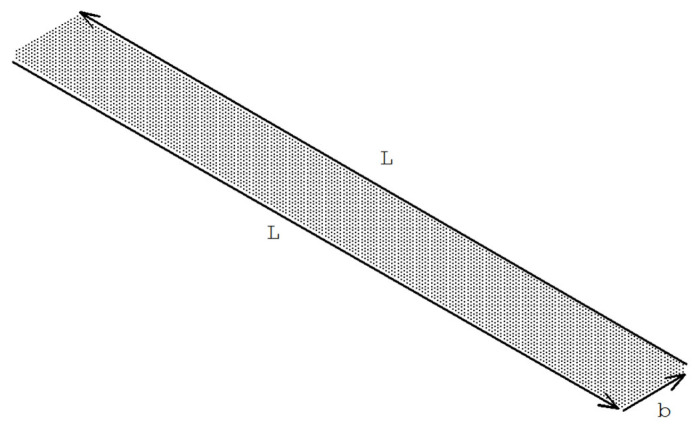
Definition of the lengthwise path (L:PATH1) and widthwise path (b:PATH2) used to extract interface stresses at the patch–core interface in the 3D model.

The geometry, the applied voltage to the PZT patches, the boundary conditions and the element size (*h* = 5 × 10^−4^ m) were kept identical in both 3D models. In addition to the tip displacement *w*(*L*), the interface stresses were examined in detail along two different paths at the patch–core interface, as illustrated schematically in Figure 6. First, a lengthwise path (L:PATH1) was defined along the patch–core interface from the fixed end to the free end in order to monitor the variation in peel $\sigma_{y}$ and shear $\tau_{xy}$ stresses in the beam axis direction. Second, a widthwise path (b:PATH2) was defined along the same interface in the transverse direction, across the patch width.

The combined PATH1 and PATH2 distributions of $\tau_{xy}$ and $\sigma_{y}$ for both element sets, together with the individual PATH2 distributions for each case, are provided in the Supplementary Materials (Figures S1–S8). These plots confirm that very high stresses are observed at the fixed end due to the idealized boundary condition; since this region does not represent a realistic delamination location, these singular values are excluded from the delamination assessment, and, the stresses along PATH1 remain relatively low away from the fixed support. The individual PATH2 distributions clearly exhibit pronounced three-dimensional edge effects and reveal the true peak interface stresses that are critical for delamination.

**Figure 7:**
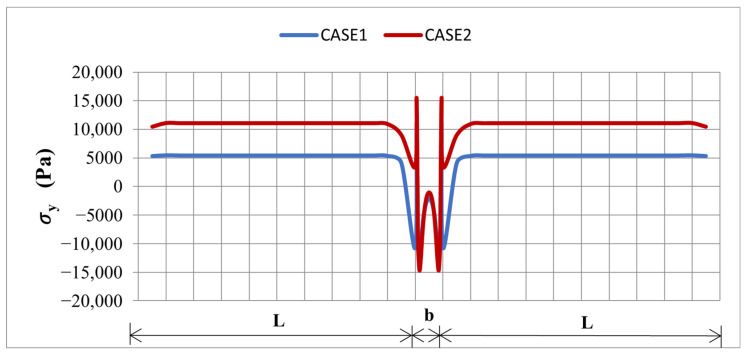
Comparison of σ_y_ along L: PATH1 and b: PATH2 for Case1 and Case 2.

**Figure 8:**
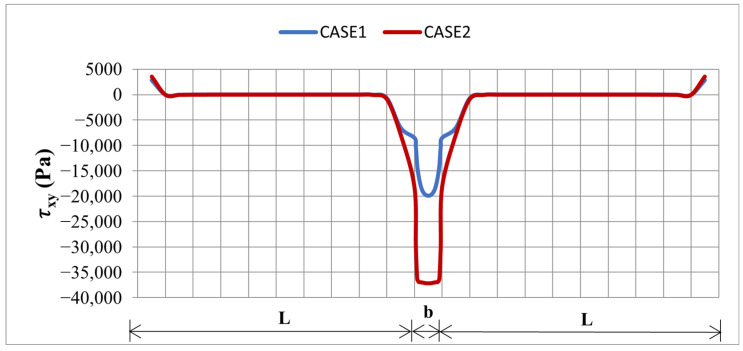
Comparison of τ_xy_ along L: PATH1 and b: PATH2 for Case1 and Case 2.

**Figure 9:**
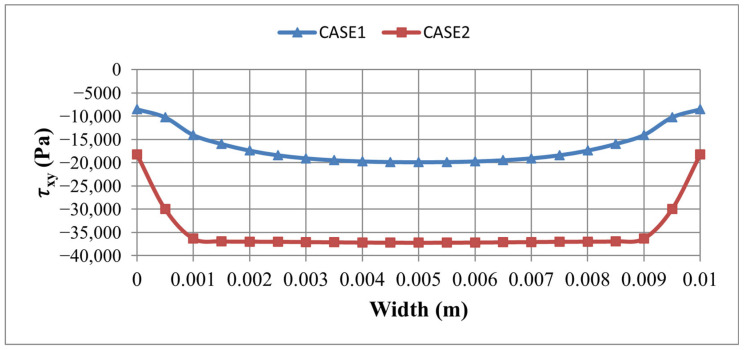
Comparison of τ_xy_ along PATH2 between Case1 and Case2.

**Figure 10:**
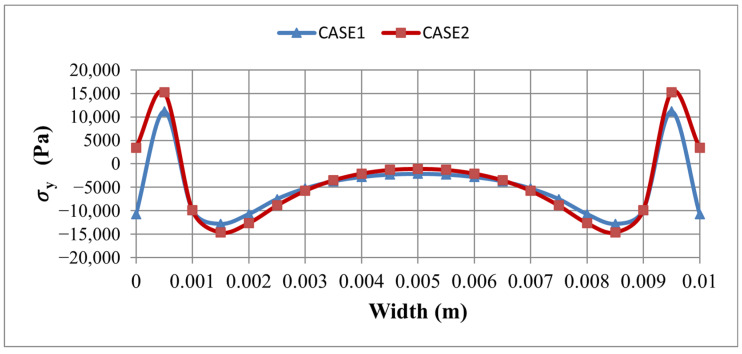
Comparison of σ_y_ along PATH2 between Case1 and Case2.

A side-by-side comparison of $\sigma_{y}$ and $\tau_{xy}$, along both PATH1 and PATH2 for the two element sets, is given in Figure 7 and Figure 8, confirming that the dominant, physically meaningful interface peaks are consistently located at the transverse edge region captured by PATH2, regardless of the element set used. A focused PATH2-only comparison between the two cases is provided in Figure 9 and Figure 10, demonstrating that: the SOLID186/SOLID226 model consistently predicts higher peak shear $\tau_{xy}$ (Figure 9) and peel $\sigma_{y}$ (Figure 10) stresses than the SOLID185/SOLID5 model, owing to its better capability to capture steep stress gradients in the thickness and edge regions. From a safety point of view, this is desirable, as it provides a more conservative estimate of the interface stress levels that drive delamination.

On the other hand, the difference in the tip displacement *w*(*L*) between the two models remains very small, indicating that the global bending stiffness is predicted almost independently of the element type, whereas the local interface stresses are significantly more sensitive to the choice of element formulation. A quantitative comparison of the tip displacement and the maximum interface peel and shear stresses obtained from the two models are summarized in Table 4.

The Case 2 solution predicts an approximately 19% higher peak peel stress $\sigma_{y}$ (from 12,865 Pa to 15,280 Pa) and an approximately 87% higher peak shear stress $\tau_{xy}$ (from 19,906 Pa to 37,213 Pa) along PATH2 compared to Case 1. This increase is attributed to the higher-order elements in Case 2 capturing the steep stress gradients near the thickness and edge regions more accurately. Although both element sets yield almost identical tip displacements, Case 2 provides a more conservative and reliable estimate of the interface stress peaks for the delamination safety assessment.

Overall, the 3D analyses demonstrate that, although low-order and high-order 3D element sets provide almost identical predictions of global tip deflection, the choice of element type has a pronounced impact on the computed interface stress peaks, which are crucial for assessing the delamination risk in patch-actuated beams.

To assess the sensitivity of the 3D stress predictions to mesh refinement, a convergence study was performed for the SOLID186/SOLID226 element set (Case 2) by varying the uniform element size *h* from 8 × 10^−4^ m to 4 × 10^−4^ m in five steps. The purpose of this study is to identify a mesh size that provides a stable and reliable prediction of both the global tip displacement and the local interface stress peaks along PATH2, which is the critical path for a delamination assessment.

**Figure 11:**
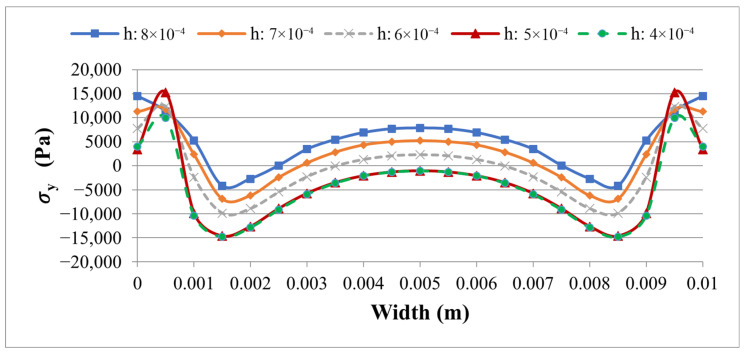
Distribution of interfacial peel stress σy along PATH2 for five mesh sizes obtained with the SOLID186/SOLID226 element set (Case 2).

**Figure 12:**
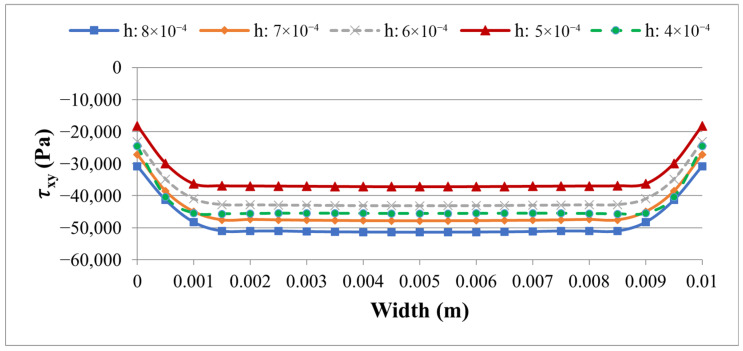
Distribution of interfacial shear stress τxy along PATH2 for five mesh sizes obtained with the SOLID186/SOLID226 element set (Case 2).

The results in Table 5 and Figure 11 and Figure 12 show that the tip displacement *w*(*L*) is completely insensitive to mesh refinement, remaining at 3.09 × 10^−7^ m across all five levels and decreasing only marginally to 3.08 × 10^−7^ m at the finest mesh (*h* = 4 × 10^−4^ m). This confirms that the global bending stiffness is well-converged throughout the entire range studied.

The interface stresses along PATH2, however, exhibit a more complex dependence on mesh size, as clearly seen in Figure 11 and Figure 12. The peak peel stress |$\sigma_{y}$| increases from 11,316 Pa at *h* = 8 × 10^−4^ m to 15,280 Pa at *h* = 5 × 10^−4^ m—a rise of approximately 35%—and then decreases slightly to 14,815 Pa at *h* = 4 × 10^−4^ m. The peak shear stress |$\tau_{xy}$| follows the opposite trend, decreasing from 51,434 Pa at *h* = 8 × 10^−4^ m to 37,213 Pa at *h* = 5 × 10^−4^ m, before rising again to 45,579 Pa at the finest mesh. This non-monotonic behavior is a well-known characteristic of localized stress concentrations at bi-material corners and free edges, where the stress field has a near-singular character and its numerical resolution is highly sensitive to the positioning of integration points relative to the concentration zone. It does not indicate a numerical instability in the model; rather, it reflects the inherent difficulty of achieving strict monotonic convergence for peak stresses at geometric discontinuities in three-dimensional finite element models.

Importantly, the mesh size *h* = 5 × 10^−4^ m yields a stable plateau in the tip displacement and represents a consistent intermediate resolution of the interface stress field, avoiding both the excessive averaging of coarse meshes and the oscillatory sensitivity of the finest discretization. This mesh size was therefore retained as the reference discretization for the comparative Case 1 vs. Case 2 study presented in Table 4. Furthermore, since the mesh sensitivity study was conducted exclusively with the higher-order SOLID186/SOLID226 element set (Case 2), which is more sensitive to the spatial resolution than the first-order SOLID185/SOLID5 set (Case 1), the chosen element size can be regarded as a conservative reference for both models.

## 6. Conclusions

Based on the thermodynamically consistent formulation and the numerical results presented in this work, the following conclusions can be drawn:

(i) Thermodynamic framework and linear piezoelectric law

A general electroelastic framework was specialized to the classical linear piezoelectric law used in finite element codes by neglecting body couples, assuming small strains and compressible elasticity, and retaining only first-order electromechanical coupling. This provides a transparent thermodynamic basis for the standard symmetric-stress piezoelectric constitutive equations and makes explicit which physical effects —finite deformation, body couples, and asymmetric stress contributions— are deliberately set aside in the present implementation.

(ii) Validation of the 2D model

The 2D finite element model was validated against the analytical Euler–Bernoulli solution and the ANSYS solution for 10 V actuation. Mesh convergence was confirmed across seven element sizes; the converged free-end displacement *w*(*L*) = 4.71 × 10^−7^ m differs from the analytical value of 4.45 × 10^−7^ m by 5.8%, a physically expected discrepancy attributable to the plane-strain constraint and the neglect of shear deformation in beam theory.

(iii) 3D interface stress characterization and PATH1/PATH2

Three-dimensional analyses with both element sets consistently show that the largest physically meaningful interface stress peaks occur along the transverse path PATH2, revealing strong three-dimensional edge effects at the lateral patch boundaries near the free end. The delamination assessment based solely on lengthwise distributions or 2D plane-strain models will miss this dominant failure-driving stress component entirely.

(iv) Effect of 3D element type on global response and interface peaks

The global tip displacement is almost insensitive to the element type (< 0.3% difference), whereas the higher-order SOLID186/SOLID226 formulation predicts an approximately 19% higher peak peel stress and 87% higher peak shear stress along PATH2 compared to SOLID185/SOLID5. This reflects the improved ability of second-order elements to resolve steep edge and through-thickness stress gradients, yielding a more conservative and physically realistic assessment of the delamination risk.

(v) Practical recommendations for model selection

For global performance assessment, first-order elements (SOLID185/SOLID5) are fully adequate and considerably more economical. For delamination risk evaluation, second-order coupled-field elements (SOLID186/SOLID226) are essential, as first-order elements can underestimate the peak interface shear stress by up to 87%. The analyses further show that the most dangerous location for delamination initiation is at the lateral free edge near the beam tip—a three-dimensional effect undetectable with 2D models. A practical two-stage workflow is recommended: a 2D model for rapid pre-screening, followed by a 3D higher-order element analysis focused on the lateral patch edge.

(vi) Limitations and future work

In this study, perfectly bonded interfaces under static actuation with a single patch configuration and a uniform voltage of 10 V were considered. Future work should address explicit adhesive layer modeling with cohesive zone elements, the activation of body couple terms through user-defined elements, the quantitative validation for alternative piezoelectric materials such as ScAlN or BaTiO_3_, and experimental validation using digital image correlation or embedded strain sensing.

## Figures and Tables

**Table 1:** Material properties of PZT-5H and Al [3].

PZT-5H											AL		
×10^8^(C^2^N^−1^m^−2^)		×10^9^(Nm^−2^)					(Cm^−2^)			(kg/m^3^)	(GPa)		(kg/m^3^)
$\epsilon_{1}^{s}$	$\epsilon_{3}^{s}$	$C_{11}^{E}$	$C_{12}^{E}$	$C_{13}^{E}$	$C_{33}^{E}$	$C_{44}^{E}$	$e_{31}$	$e_{33}$	$e_{15}$	ρ	Y	υ	ρ
1.504	1.301	126	79.5	84.1	117	23	−6.5	23.3	17	7500	70	0.33	2800

**Table 2:** Displacements at the free end according to element dimensions.

*h* (m)	Number of Elements	Number of Nodes	*w*(*L*) (m)	r (%)
5 × 10^−3^	120	147	4.27 × 10^−7^	-
2.5 × 10^−3^	360	410	4.44 × 10^−7^	3.981
1 × 10^−3^	1800	1919	4.59 × 10^−7^	3.378
5 × 10^−4^	7200	7437	4.65 × 10^−7^	1.307
2.5 × 10^−4^	28,800	29,273	4.68 × 10^−7^	0.645
1 × 10^−4^	180,000	181,181	4.71 × 10^−7^	0.641
5 × 10^−5^	720,000	722,361	4.71 × 10^−7^	0

**Table 3:** Results and percent error.

*h* (m)	*w*(*L*)_ANSYS_ (m)	*w*(*L*)_Analytical_ (m)	% Error
5 × 10^−3^	4.27 × 10^−7^	4.45 × 10^−7^	% 4.04
2.5 × 10^−3^	4.44 × 10^−7^	4.45 × 10^−7^	% 0.22
1 × 10^−3^	4.59 × 10^−7^	4.45 × 10^−7^	% 3.15
5 × 10^−4^	4.65 × 10^−7^	4.45 × 10^−7^	% 4.49
2.5 × 10^−4^	4.68 × 10^−7^	4.45 × 10^−7^	% 5.17
1 × 10^−4^	4.71 × 10^−7^	4.45 × 10^−7^	% 5.84
5 × 10^−5^	4.71 × 10^−7^	4.45 × 10^−7^	% 5.84

**Table 4:** Results of Case 1 and Case 2 solutions.

Element Set	Beam Element	PZT Element	Node Order	w(L) (m)	$\mathrm{Max}\;\left|\sigma_{y}\right|$ (Pa)	$\mathrm{Max}\;\left|\tau_{xy}\right|$ (Pa)
Case-1	SOLID185	SOLID5	1-degree (8-node)	3.10 × 10^−7^	12,865	19,906
Case-2	SOLID186	SOLID226	2-degree (20-node)	3.09 × 10^−7^	15,280	37,213

**Table 5:** Effect of mesh size on tip displacement *w*(*L*), peak peel stress $\sigma_{y}$, and peak shear stress $\tau_{xy}$ along PATH2 for the SOLID186/SOLID226 (Case 2) 3D model.

*h* (m)	*w*(*L*) (m)	$\mathrm{Max}\;\left|\sigma_{y}\right|$ (Pa)	$\mathrm{Max}\;\left|\tau_{xy}\right|$ (Pa)
8 × 10^−4^	3.09 × 10^−7^	11,316	51,434
7 × 10^−4^	3.09 × 10^−7^	11,706	47,876
6 × 10^−4^	3.09 × 10^−7^	12,320	43,172
5 × 10^−4^	3.09 × 10^−7^	15,280	37,213
4 × 10^−4^	3.08 × 10^−7^	14,815	45,579

## Data Availability

The original contributions presented in this study are included in the article/Supplementary Materials. Further inquiries can be directed to the corresponding author.

## Supplementary Materials

The following supporting information can be downloaded at: https://www.mdpi.com/article/10.3390/ma19091864/s1. Figure S1. Comparison of interfacial shear stress τ_xy_ along PATH1 and PATH2 for Case 1. Figure S2. Comparison of interfacial peel stress σ_y_ along PATH1 and PATH2 for Case 1. Figure S3. Distribution of interfacial shear stress τ_xy_ along PATH2 for Case 1. Figure S4. Distribution of interfacial peel stress σ_y_ along PATH2 for Case 1. Figure S5. Comparison of interfacial shear stress τ_xy_ along PATH1 and PATH2 for Case 2. Figure S6. Comparison of interfacial peel stress σ_y_ along PATH1 and PATH2 for Case 2. Figure S7. Distribution of interfacial shear stress τ_xy_ along PATH2 for Case 2. Figure S8. Distribution of interfacial peel stress σ_y_ along PATH2 for Case 2.

## Nomenclature

The bold characters represent vectors and tensors. Summation is implied over repeated indices (Einstein summation convention) [25,28]:

Latin Letters	
B	Body force per unit mass (gravitational force)
b	Width of the beam
$C_{LN}$	Green deformation tensor
${\dot{C}}_{LN}$	Deformation (strain) rate tensor in material coordinates
D	Electric displacement vector
$d_{31}$	Piezoelectric strain coefficient
$d_{ij}$	Deformation (strain) rate tensor
dS	Surface area element
dV	Volume element
E	Electric field vector
$E_{LN}$	Green–Lagrange strain tensor (finite deformation)
${\dot{E}}_{LN}$	Green–Lagrange strain rate tensor
${\tilde{E}}_{IJ}$	Strain tensor in the linearized theory
${\tilde{e}}_{ij}$	Infinitesimal strain tensor in spatial coordinates
$F_{P}$	Distributed force due to polarization and electric field
$F_{mM}$	Deformation gradient of the forward motion
$F_{Ii}^{-1}$	Deformation gradient of the inverse motion
$G_{M}$	Temperature gradient in material coordinate
h	Heat energy source per unit mass
$h^{E}$	Electrostatic energy source
I	Second moment of area of the cross-section
J	Jacobian
$K_{0}$	Curvature
L	Beam length
$m_{P}$	Distributed couple due to polarization and electric field
M	Bending moment
n	Outward unit normal vector of the discontinuity surface
P	Polarization vector
q	Heat flux vector
$Q_{I}$	Heat vector in material coordinates
r(h)	Successive difference ratio (convergence criterion)
S(t)	Surface of the body
t	Time
$t_{c}$	Thickness of the aluminum core
$t_{p}$	Thickness of the PZT patch
u	Displacement vector
$u_{i}$	Components of the displacement vector
U	Relative displacement velocity of the discontinuity surface with respect to the medium
V	Applied electric potential
V(t)	Volume
v	Velocity vector
$v_{i,j}$	Velocity gradient tensor
w(L)	Free-tip displacement
$w_{f}$	Free surface charge distribution
$w_{ij}$	Spin (whirl) tensor
X	Position vector in the undeformed configuration
$X_{M,m}$	Deformation gradient of inverse motion
y	Position vector in the deformed configuration
$y_{m,M}$	Deformation gradient
Y	Young’s modulus
YI	Bending stiffness per unit width
$z_{t},z_{b}$	Thickness coordinate for the upper and lower patches
Greek Letters	
$\Sigma_{ijkl}$	Elasticity tensor components in spatial coordinates
$\mu_{ijl}$	Piezoelectric coupling tensor components in spatial coordinates
$\beta_{il}$	Electric permittivity tensor components in spatial coordinates
$\epsilon_{ij}$	Infinitesimal strain tensor components
$\xi_{0}$	Permittivity of free space
η	Entropy density per unit volume
ε	Internal energy per unit volume
θ	Temperature
Π_I_	Polarization vector per unit mass
ρ	Mass density in the deformed configuration
$\rho_{0}$	Mass density in the reference configuration
Σ	Thermodynamic stress potential
$\sigma_{ij}$	Asymmetric stress tensor components
${\bar{\sigma }}_{ij}$	Symmetric stress tensor components
$\sigma_{y}$	Peel stress
$\tau_{xy}$	Interface shear stress
φ	Electric potential
ψ	Helmholtz free energy density/thermodynamic potential
Γ	Discontinuity surface
$\omega_{k}$	Angular velocity components
Special Notation	
$\delta_{ij}$	Kronecker delta
$\varepsilon_{ijk}$	Permutation tensor
∇	Gradient operator
${\left(\cdot \right)}_{i}$	Derivative with respect to variable i
$⟦\cdot ⟧$	Jump across the discontinuity surface
PATH1	Longitudinal interface path (clamped end → free end)
PATH2	Transverse interface path (along patch width)
Abbreviations	
FEM	Finite Element Method
PZT	Lead–Zirconate–Titanate
DOF	Degree of Freedom
APDL	ANSYS Parametric Design Language
2D/3D	Two-Dimensional/Three-Dimensional
