# Supplementary material for: Thermodynamically Consistent Linear Electroelastic Formulation and FEM Study of Patch-Actuated Smart Structures: Validation and Interface Stress Evaluation
Source: Materials (Basel). 2026 May 1;19(9):1864. doi: 10.3390/ma19091864 (PMC13164608; doi:10.3390/ma19091864)
Supplement: Supplementary file 1 [file materials-19-01864-s001.zip › materials-4238780-supplementary.pdf]

Supplementary Information

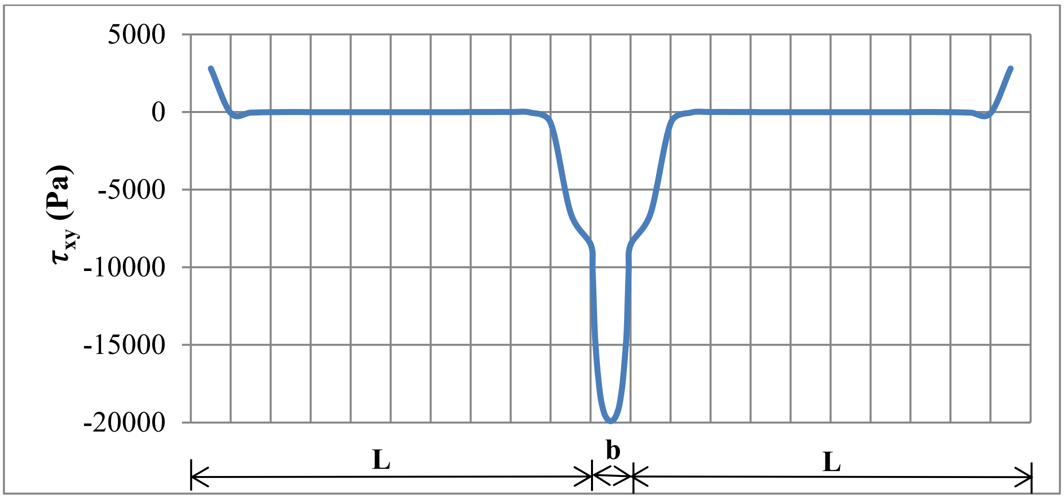

Figure S1. Comparison of interfacial shear stress τ_{xy} along PATH1 and PATH2 for Case 1.

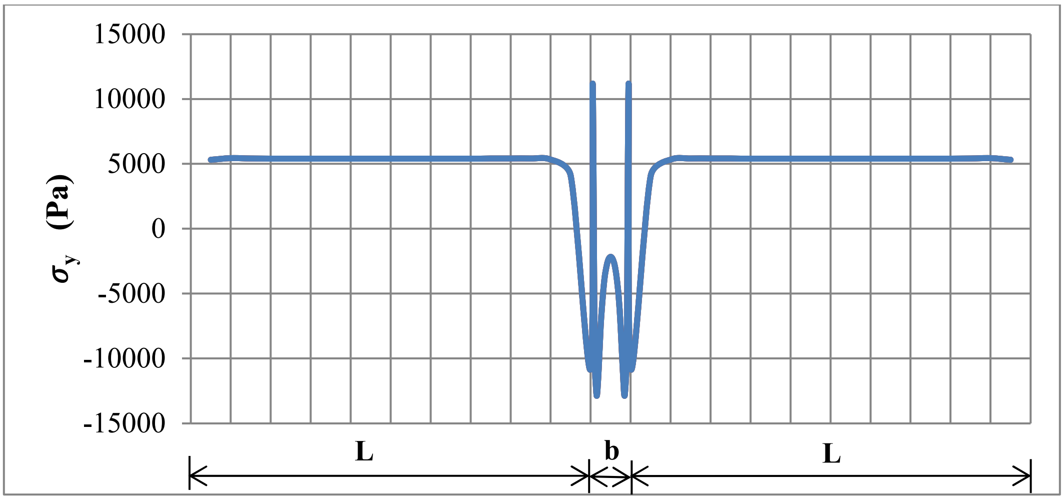

Figure S2. Comparison of interfacial peel stress σ_y along PATH1 and PATH2 for Case 1.

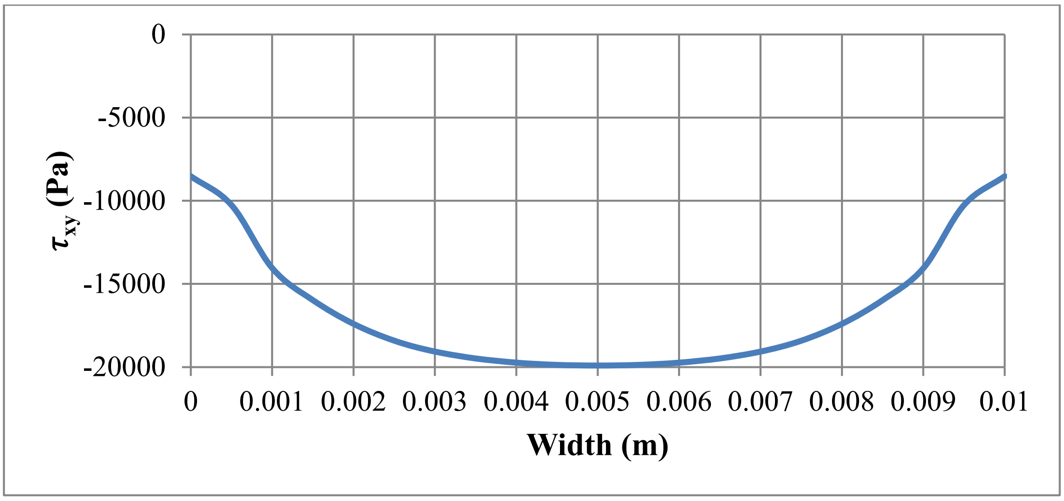

Figure S3. Distribution of interfacial shear stress τ_{xy} along PATH2 for Case 1.

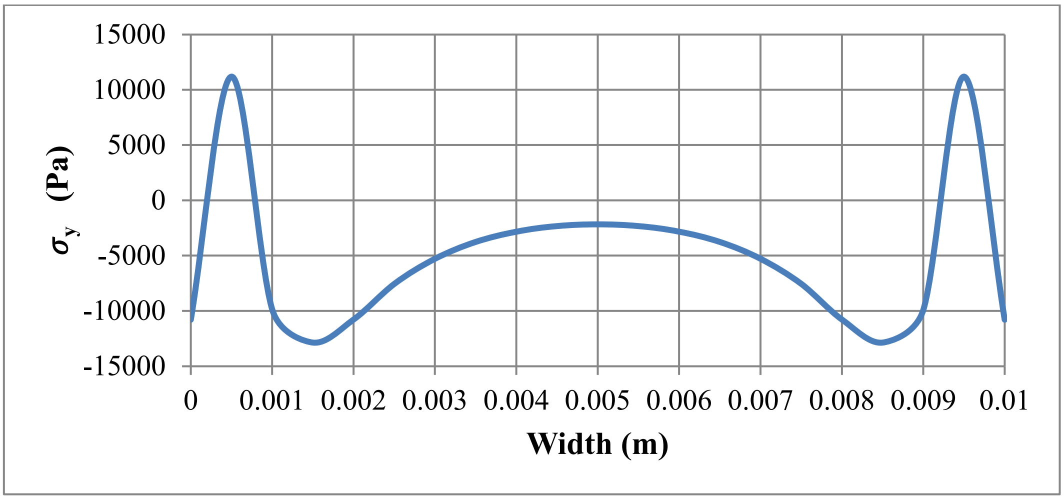

Figure S4. Distribution of interfacial peel stress σ_y along PATH2 for Case 1.

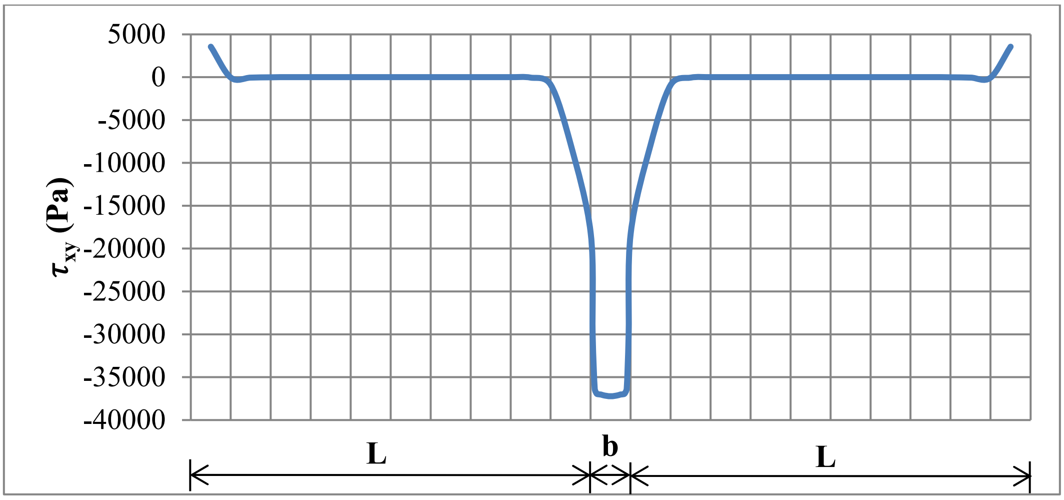

Figure S5. Comparison of interfacial shear stress τ_{xy} along PATH1 and PATH2 for Case 2.

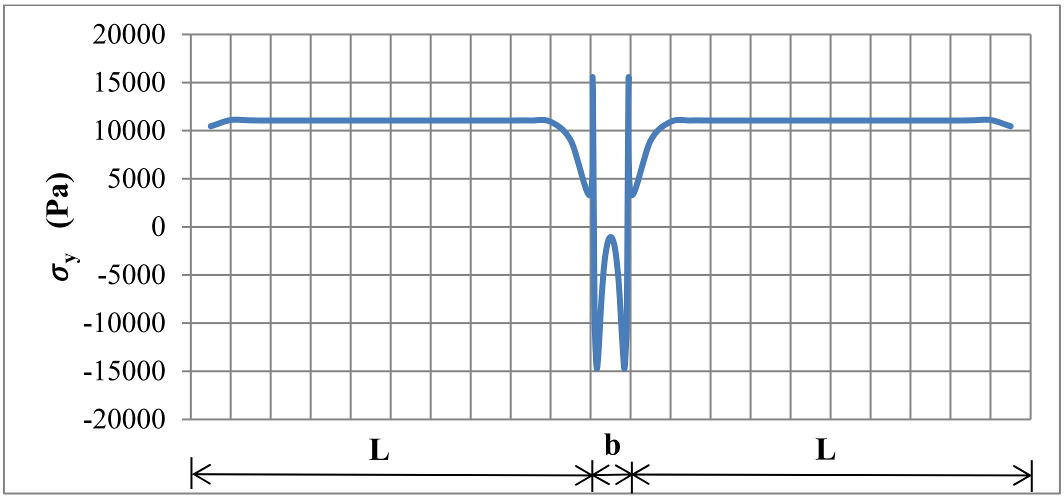

Figure S6. Comparison of interfacial peel stress σ_y along PATH1 and PATH2 for Case 2.

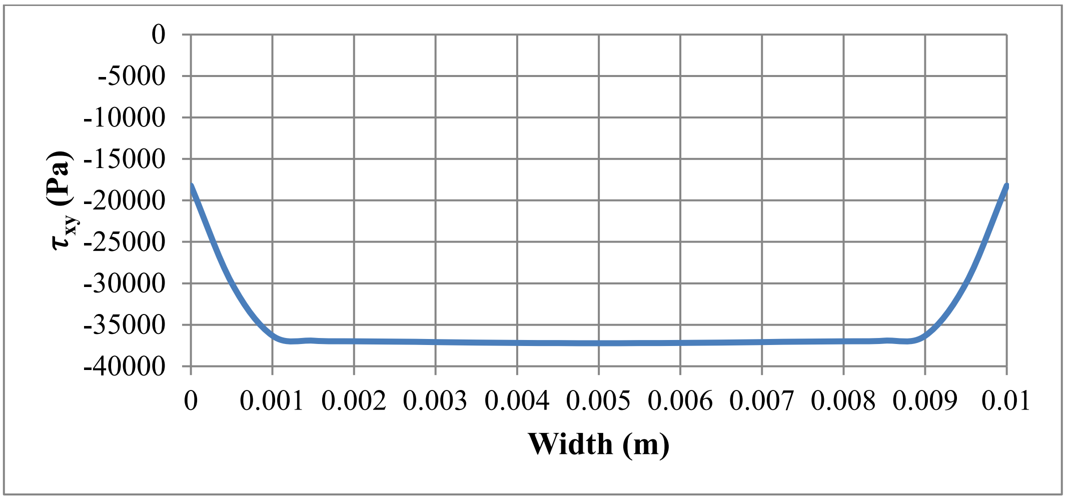

Figure S7. Distribution of interfacial shear stress τ_{xy} along PATH2 for Case 2.

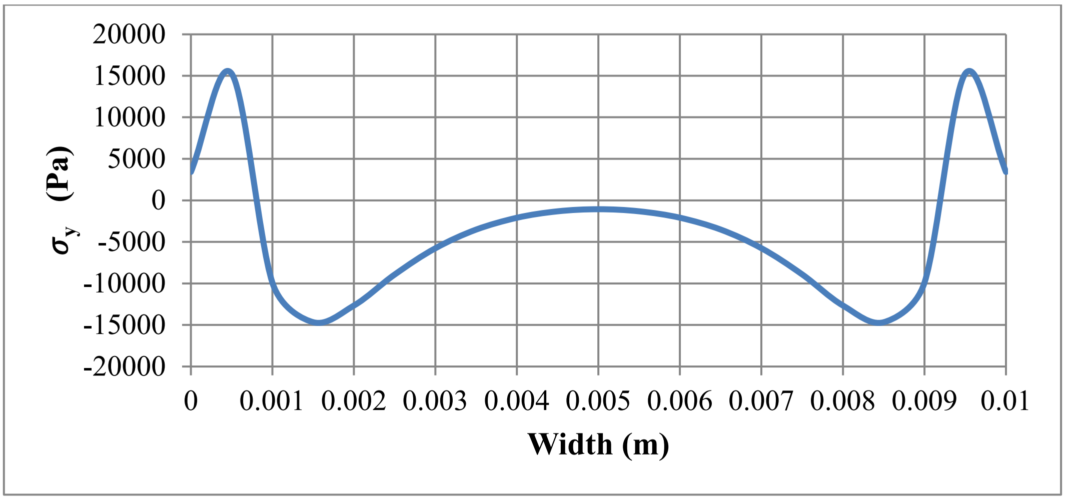

Figure S8. Distribution of interfacial peel stress σ_y along PATH2 for Case 2.
